# Nanomechanical and Microstructural Characterization of Biocompatible Ti**_3_**Au Thin Films Grown on Glass and Ti_**6**_Al_4_V Substrates

**DOI:** 10.1021/acsbiomaterials.4c00070

**Published:** 2024-04-17

**Authors:** Cecil Cherian Lukose, Ioannis Anestopoulos, Mihalis I. Panayiotidis, Martin Birkett

**Affiliations:** † Department of Mechanical and Construction Engineering, Northumbria University, Ellison place, Newcastle upon Tyne NE1 8ST, U.K.; ‡ Department of Cancer Genetics, Therapeutics & Ultrastructural Pathology, The Cyprus Institute of Neurology and Genetics, Nicosia 1683, Cyprus

**Keywords:** nanoindentation, hardness, sputtering, biocompatible, Ti_3_Au thin film coating, L929 mouse fibroblasts

## Abstract

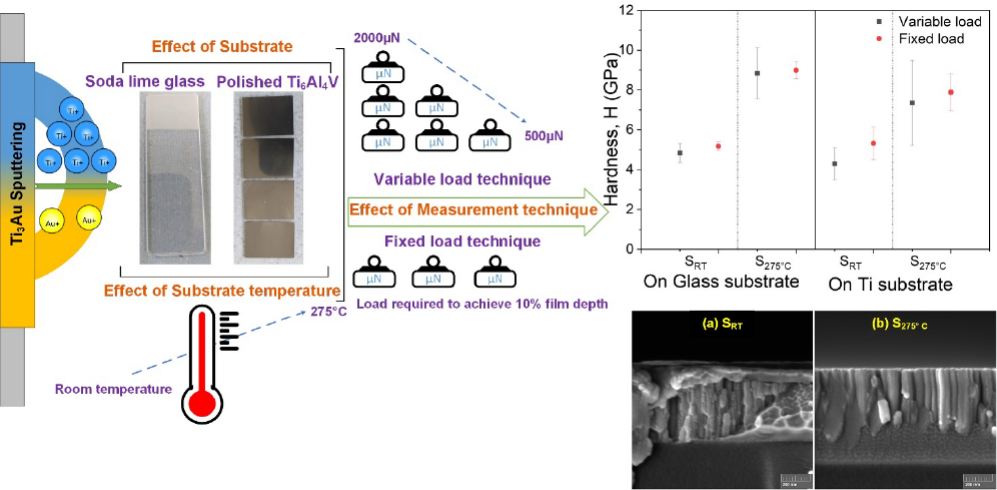

Ti–Au intermetallic-based material systems are being extensively studied as next-generation thin film coatings to extend the lifetime of implant devices. These coatings are being developed for application to the articulating surfaces of total joint implants and, therefore, must have excellent biocompatibility combined with superior mechanical hardness and wear resistance. However, these key characteristics of Ti–Au coatings are heavily dependent upon factors such as the surface properties and temperature of the underlying substrate during thin film deposition. In this work, Ti_3_Au thin films were deposited by magnetron sputtering on both glass and Ti_6_Al_4_V substrates at an ambient and elevated substrate temperature of 275 °C. These films were studied for their mechanical properties by the nanoindentation technique in both variable load and fixed load mode using a Berkovich tip. XRD patterns and cross-sectional SEM images detail the microstructure, while AFM images present the surface morphologies of these Ti_3_Au thin films. The biocompatibility potential of the films is assessed by cytotoxicity tests in L929 mouse fibroblast cells using Alamar blue assay, while leached ion concentrations in the film extracts are quantified using ICPOEMS. The standard deviation for hardness of films deposited on glass substrates is ∼4 times lower than that on Ti_6_Al_4_V substrates and is correlated with a corresponding increase in surface roughness from 2 nm for glass to 40 nm for Ti_6_Al_4_V substrates. Elevating substrate temperature leads to an increase in film hardness from 5.1 to 8.9 GPa and is related to the development of a superhard β phase of the Ti_3_Au intermetallic. The standard deviation of this peak mechanical hardness value is reduced by ∼3 times when measured in fixed load mode compared to the variable load mode due to the effect of nanoindentation tip penetration depth. All tested Ti–Au thin films also exhibit excellent biocompatibility against L929 fibroblast cells, as viability levels are above 95% and leached Ti, Al, V, and Au ion concentrations are below 0.1 ppm. Overall, this work demonstrates a novel Ti_3_Au thin film system with a unique combination of high hardness and excellent biocompatibility with potential to be developed into a new wear-resistant coating to extend the lifetime of articulating total joint implants.

## 1 Introduction

Titanium (Ti) and its alloys like Ti_6_Al_4_V are widely used in total joint implant applications, such as knee, hip, shoulder, and elbow joints, because of their excellent biocompatibility, corrosion resistance, strength to weight ratio, osseointegration, and low ion formation in aqueous media.^1−4^ However, these alloys exhibit poor wear performance when subjected to repetitive articulating motion in load bearing joints, leading to the unwanted release of aluminum and vanadium particles, which are highly toxic and have been linked to numerous adverse health effects.^5,6^ Increased failure of implants made from these alloys has led to an increase in reconstruction surgery at heavy financial cost.^5−8^ Therefore, a suitable coating system, which can enhance the mechanical performance while maintaining the excellent biocompatibility of Ti, is being extensively researched.^9^

Recently, Ti–Au intermetallics have been found to exhibit excellent biocompatibility combined with extremely high mechanical hardness with the emergence of the superhard β-Ti_3_Au intermetallic phase.^10^ Svanidze et al. found that bulk samples of Ti–Au alloy formed by an arc melting process exhibit a monotonous increment in mechanical hardness with increasing gold (Au) concentration, reaching a peak value of 800 HV (∼7.85 GPa), when approaching a stoichiometric ratio of Ti_3_Au.^11^ Au has a dense valence electron arrangement compared to other biocompatible elements, which leads to very high mass density, and when alloyed with Ti, this causes an increased bond strength, which in turn leads to higher hardness.^11^ The Ti_3_Au intermetallic exists in two distinctive phases, denoted as alpha (α) and beta (β).^12^ The α phase has a smaller unit cell (lattice parameter ∼4.1 Å) with Ti atoms arranged in 12-fold coordination, whereas the β phase has a larger unit cell (∼5.1 Å) with Ti atoms existing in 14-fold coordination, making it much denser. The higher density packing of the β phase presents a higher energy barrier for slipping of dislocations, thereby resulting in higher hardness.^10−14^ These factors lead to enhanced hardness and low coefficient of friction for the β-Ti_3_Au intermetallic, making it an ideal candidate as a wear-resistant coating over implants. Thin film depositions of Ti–Au and Ti_3_Au intermetallics have mostly been carried out on silicon (Si) substrates. Silicon was preferred as substrate because the surface is extremely smooth and the properties of Si are well-known. which allows the substrate background to be easily removed, for example, when analyzing thin films using X-ray characterization techniques or performing an AFM scan.^12,14^ Recently, Karimi and Cattin achieved an elastic modulus of 201 GPa and a mechanical hardness of 12.5 GPa for Ti_3_Au thin films deposited on Si substrates.^12^ However, to gain a realistic understanding of Ti_3_Au intermetallic coatings, it is critical that their performance is studied on real-world substrate materials like Ti_6_Al_4_V, that is used to manufacture total knee and hip prosthesis.^15^

In our previous preliminary work,^16^ we demonstrated that Ti–Au thin films with superior mechanical performance and excellent biocompatibility can be achieved by carefully controlling the Ti and Au atomic ratio and thermal activation process. In the current work, we study the effect of the underlying substrate type and its surface conditions and temperature on the mechanical performance and biocompatibility of Ti_3_Au thin film coatings sputter deposited on glass and Ti_6_Al_4_V substrates. Thin film samples on glass are used for accurate analysis of the elemental composition and microstructure pattern, while those deposited on Ti_6_Al_4_V substrates under the same conditions allow us to understand the potential of Ti_3_Au as a mechanically hard, biocompatible thin film coating. This work also explores the effect of the nanoindentation measurement technique employed to measure the mechanical properties of Ti_3_Au thin films, helping to isolate the substrate and surface size effect. Both variable load and fixed load protocols were applied to understand the measured mechanical properties. Therefore, this paper strives to cover the void in understanding the effect of substrate type and temperature and measurement technique on the combination of mechanical and biocompatibility properties of Ti_3_Au intermetallic thin film coatings with the potential to extend the lifetime of the articulating surfaces of total joint implants.

## 2 Materials and Methods

### 2.1 Thin Film Deposition

Sputter deposition of Ti_3_Au thin films was performed by using a Moorefield NanoPVD deposition suite. The chamber was loaded with 2-inch diameter circular targets of Ti and Au of 99.999% purity supplied by Pi-Kem limited, UK, with the Ti target connected to a DC source and the Au target to a RF source. Laboratory-grade soda lime glass slides and commercially procured Ti_6_Al_4_V strips measuring 76 × 26 mm and a thickness of 1 mm were used as substrates. The Ti_6_Al_4_V substrates were rigorously polished using SiC paper with grit values of 240, 320, 600, 1200, and 4000 to achieve a mirror-like surface finish with roughness values better than 40 nm, when measured using an Alicona Infinity Focus surface measurement system. The polished Ti_6_Al_4_V substrates were cut into 4 rectangular coupons, each measuring 25 mm × 19 mm, before being thoroughly cleaned, together with the glass substrates, using a DECON 90 surface cleaner in a 5:1 ratio with water, followed by an ultrasonic bath in DI water, IPA cleaning, and acetone wiping and a second ultrasonic bath in DI water, before finally being dried with a jet of nitrogen. The cleaned substrates were loaded onto the deposition plant substrate holder at a target to substrate distance of 100 mm and rotated at a constant speed of 5 rpm, and then the chamber was evacuated to a base pressure better than 5 × 10^–4^ Pa. For the sputtering runs, a constant working pressure of 0.6 Pa was achieved by introducing 10 sccm of Ar gas in the chamber and the DC to RF power ratio required to achieve a 3:1 ratio of Ti:Au was established. It is known that the β-phase of Ti_3_Au crystallizes better at higher substrate temperature,^12^ and therefore, two sets of samples were deposited: one with the substrate temperature set to ambient (∼25°C) and the second one with the substrate heater set to achieve 275 °C on the substrate surface.

### 2.2 Structural, Morphological, and Mechanical Characterizations

The crystal structure of the deposited Ti_3_Au films was characterized by the X-ray diffraction technique using a Rigaku Smartlab II diffractometer, employing Cu Kα radiation in a parallel beam configuration. The reflection patterns were collected between 2θ values of 10 and 80° with a step size of 0.01° and a scan rate of 4°/min. The peaks were indexed using the supplied database and cross-referenced with the files from the ICSD database. Surface and cross-sectional features of the deposited thin films were captured using a MIRA III scanning electron microscope (SEM) from TESCAN Systems, operating at 5 kV and a close working distance of 5 mm from the tip of the e-beam lens. An X-Max 150, energy dispersive X-ray (EDX) spectroscopy detector from Oxford Instruments, in-built within the SEM, was used to analyze the elemental composition of the deposited thin films. Surface scans were performed on a 3 μm^2^ area of the thin films using a Nanoveeco Dimension 3000 atomic force microscope (AFM), and the scans were analyzed using Gwyddion software to measure the surface roughness and feature sizes. Nanoindentations were performed by a Hysitron TI900 triboindenter nanomechanical testing system, employing a 3-sided Berkovich diamond tip. Two sets of indentations were performed: one with a variable load and the other with a constant load. For the first set, the load was varied from 2000 to 500 μN in steps of 100 μN. For the second set, the load was kept at a constant value to achieve a total indentation depth of 10% of the thin film thickness under test. For each sample, 16 indents were made in a 4 × 4 pattern, with a 10 μm gap between each indent and a 10–10–10 s load–dwell–unload segment time. Following Oliver and Phàrr’s methodology,^17,18^ the force–displacement curve was plotted and the unloading segment was analyzed to extract mechanical hardness and elastic modulus values of the thin films. The average value of the 16 indents for each sample is presented with the standard deviation.

### 2.3 Cytotoxicity and Biocompatibility Analyses

The biocompatibility of the deposited Ti_3_Au thin films was analyzed in accordance with the ISO 10993 standard by measuring their in vitro cytotoxicity and ion leaching potential. L929 cells (murine fibroblasts) were acquired from Deutsche Sammlung von Microorganismen and Zellkulturen (DSMZ – Braunschweig, Germany) and cultured in Dulbeccos’s Modified Eagle Medium (DMEM), high glucose, supplemented with 10% fetal bovine serum (FBS), 2 mM l-glutamine, 100 U/mL penicillin, and 100 μg/mL streptomycin. L929 cells were cultured under humidified conditions at 37 °C and 5% CO_2_, grown as monolayer cultures. When confluency reached 80–90%, cells were subcultured for a maximum of 20–25 passages, before new vials were used. Cell culture media and reagents [FBS, antibiotics, trypsin, l-glutamine, phosphate buffer saline (PBS)] were procured from Biosera (Kansas City, MO, USA). Resazurin sodium salt was obtained from Fluorochem (Derbyshire, UK), and cell culture plastic ware was supplied by Corning (NY, USA). Ti_3_Au coating extracts were prepared by immersing the thin film test coupons into 6-well plates containing 6 mL of DMEM culture media for 72 h in a humidified incubator at 37 °C and in 5% CO_2_. A second set of extracts were created by incubating coupons for additional 96 h (total 168 h), before the leached culture media were used for cytotoxicity tests against L929 cells. Similarly, extracts were also prepared from a blank polished Ti_6_Al_4_V substrate, as well as from a polished copper (Cu) substrate of similar size, used as negative and positive cytotoxicity controls, respectively. A light agitation at the beginning and the end of leaching periods (72 and 168 h) was performed in six-well plates containing the Ti_3_Au films, in order to efficiently obtain ion leaching, before their use in cytotoxicity experiments.

The cytotoxicity profile of the Ti_3_Au films on L929 mouse fibroblast cells was tested by using the Alamar blue Assay. Specifically, L929 cells were seeded at a density of 2000 cells/well in 100 μL/well into 96-well plates and left overnight to attach. The following day DMEM cell culture media were removed, and the cells were incubated with culture media containing extracts from the Ti_3_Au films, following either 72 or 168 h leaching periods, as previously described, for a total of 72 h. Complete DMEM media (Control), as well as leached media from the blank Ti_6_Al_4_V substrate, were used as negative controls, while Cu substrate leached extracts and 10% DMSO were used as positive control samples. At the end of 72 h exposures, 10 μL of resazurin (1 mg/mL final concentration) was added to each well, and cells were incubated in a humidified incubator for 4 h at 37 °C and 5% CO_2_. Finally using an absorbance plate reader (Labtech LT4500, UK), absorbance measurements were performed at 570 and 590 nm (reference wavelength) and optical density was measured as the difference between the intensity measured at 570 versus 590 nm, while cell viability levels were calculated and expressed as a percentage (%) of untreated (BLANK, control) cells.

The remaining quantities of extracts prepared from the Ti_3_Au films and Cu positive control substrates were tested for leached ion concentrations using a PerkinElmer Optima 8000 inductively coupled plasma optical emission mass spectrometer (ICP-OEMS). Standards were prepared for the range of 1–10 ppm to identify dissolved concentrations of Ti, Al, V, Cu, and Au ions leaching out from the underlying Ti_6_Al_4_V substrate, Ti_3_Au thin films, or the Cu positive control.

## 3 Results and Discussion

### 3.1 Chemical and Structural Results

The results from the elemental composition analysis and cross-sectional film thickness measurements are presented in Table 1. Thin films deposited at room temperature (S_RT_) and elevated substrate temperature of 275 °C (S_275 °C_) both exhibit Ti:Au composition very close to the required 75:25 at% ratio. This composition is shown to be most ideal for the development of the β phase of the Ti_3_Au intermetallic.^12^ The thickness of films, measured from the cross-sectional images in Figure 3, show that the thin film deposited at room temperature registers a thickness of 533 nm compared to 676 nm for the film deposited at a substrate temperature of 275 °C. This increment of over 140 nm in film thickness at higher temperatures can be associated with the grain growth taking place in the film microstructure when higher adatom energy is provided by thermal processes like substrate heating and heat treatment.^19−21^

**Table 1:** The Film Thickness and Elemental Composition of Ti_3_Au Thin Film Samples

sample ID	film thickness in nm [std. dev]	Ti:Au elemental composition in at % [std. dev]
S_RT_	533 [±13]	74.9:25.1 [±1.4]
S_275 °C_	676 [±15]	74.1:25.9 [±1.4]

**Figure 1:**
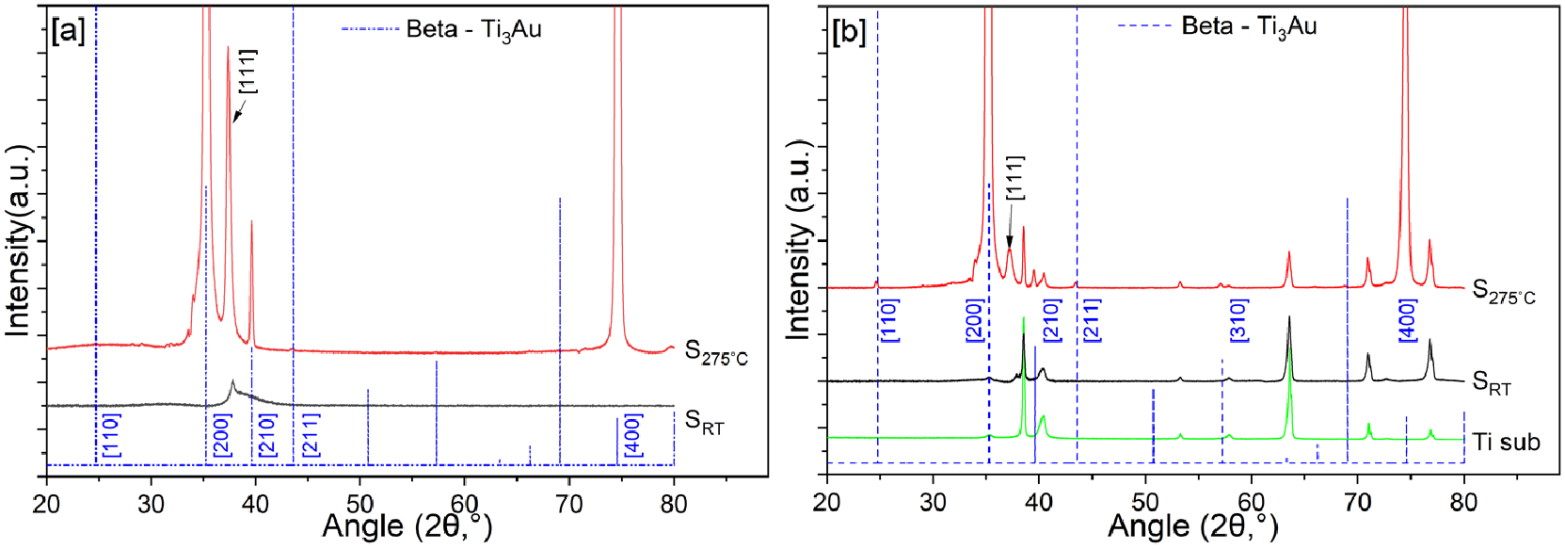
Diffraction patterns of Ti_3_Au thin films deposited on (a) glass and (b) a Ti_6_Al_4_V substrate.

The microstructure of the deposited Ti_3_Au films was studied by X-ray diffraction, and the resulting reflection patterns are presented in Figure 1. Figure 1a presents the diffraction patterns for thin films deposited on glass substrates with and without substrate heating, compared against standard peak positions for the β phase of the Ti_3_Au intermetallic (dashed blue line) from the ICSD (collection no.58605). The thin film sample deposited without substrate temperature S_RT_ (black line) is seen to represent a quasi-crystalline structure with a very broad peak spanning from 36 to 42°, with its peak positioned at 37.8°. It is known from ICSD (collection no. 58604) that the (111) plane of α-Ti_3_Au has its peak positioned at 37.5°, so we can assume that some strained Ti_3_Au intermetallic phase begins to emerge for this sample, but the energy associated with adatoms in the absence of any thermal process is not sufficient to form well-crystallized peaks.^22^ However, for sample S_275 °C_, deposited at an elevated substrate temperature of 275 °C, it can be seen that crystallization improves drastically with very sharp peaks. The peaks located at 35.27°, 39.6°, and 74.57° align very well with the (200), (210), and (400) planes of β-Ti_3_Au, whereas the peak at 37.5° suggests the coexistence of the (111) plane of α-Ti_3_Au. The elevated substrate temperature increases the energy associated with adatoms arriving on the substrate surface and helps them to diffuse effectively, leading to better crystallization of the growing thin film.^23^Figure 1b presents the X-ray characterization of Ti_3_Au thin films deposited on Ti_6_Al_4_V substrates, together with the background reflections expected from the underlying blank Ti_6_Al_4_V substrate (green line) and the expected peak positions for the β-Ti_3_Au intermetallic phase. By comparing the reflections from the bare Ti_6_Al_4_V substrate and the thin film grown without substrate temperature, S_RT_, it can be clearly seen that this sample does not register any peaks, other than broadening of the peak at 38.5°, which originates from the Ti_6_Al_4_V substrate. This peak broadening again suggests that the thin films deposited on Ti_6_Al_4_V substrates without substrate heating also exhibit a quasi-crystalline nature. Similar to the results on glass substrates, the thin film samples deposited at an elevated substrate temperature of 275 °C (S_275 °C_) exhibit very clear peak positions belonging to the α and β phases of the Ti_3_Au intermetallic, in addition to the reflection peak originating from the underlying Ti_6_Al_4_V substrate, showing that improved crystallization occurs with higher adatom energies.

### 3.2 Morphological Results

**Figure 2:**
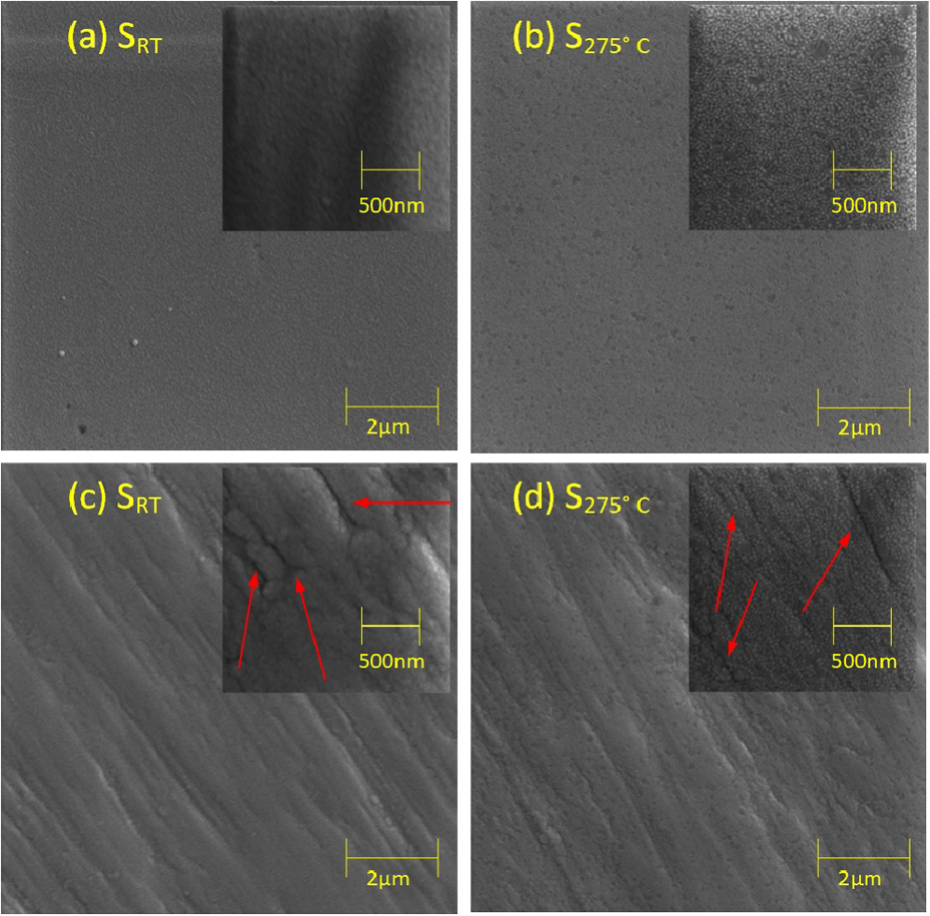
Surface morphology of Ti_3_Au thin films deposited on glass substrate at (a) room temperature and (b) substrate temperature of 275 °C and on Ti_6_Al_4_V substrate at (c) room temperature and (d) substrate temperature of 275 °C [higher magnification image of each sample provided in the inset].

Surface images of Ti_3_Au films deposited on glass substrates, with and without substrate heating, are presented in Figure 2,b, respectively, along with their high magnification version in the inset. The surface of the thin film sample deposited without additional substrate heating, S_RT_, has a smooth glass-like texture with a very fine random structure. This type of surface texture is typical of poorly crystallized microstructures and is in agreement with the very broad peak seen in the XRD pattern for this film (Figure 1a).^22^ The presence of very fine and randomly distributed structures can be related to alignment of the XRD peak centralized at 37.8°, and together, these results strengthen the emergence of the α phase of Ti_3_Au when thin films are deposited in the correct stoichiometry, even without substrate heating. However, Figure 2b shows that the thin film deposited on glass at elevated substrate temperature, S_275 °C_, has well-organized, oval shaped grains, distributed uniformly throughout most of the film surface, [better visualized in higher magnification image in Figure 2b inset]. This pattern of oval grains is broken by intermediate patches of glass-like texture, as seen before for sample S_RT_. The presence of oval shaped grains on the surface of S_275 °C_ can be correlated with the emergence of the sharp XRD peaks representing β-Ti_3_Au seen for this film (Figure 1a), while the patches of featureless regions can be assigned to the sparsely distributed α phase of Ti_3_Au, which also appears in the XRD pattern as a single peak at 37.5°.

Figure 2c,d shows the surfaces of the Ti_3_Au film samples deposited on Ti_6_Al_4_V substrates with and without substrate temperature, respectively. A key difference in these images compared to their glass counterparts is the presence of large polishing grooves (indicated by red arrows in Figure 2c,d) across the surface of the Ti_6_Al_4_V substrates. Even though the surface was polished to a mirror finish with surface roughness values better than 40 nm, the Ti_6_Al_4_V substrates are still very rough when compared to glass, which has typical roughness values of less than 2 nm. Apart from these polishing grooves, the surface features of the thin films deposited on Ti_6_Al_4_V substrates look very similar to those on glass. The thin films deposited without substrate temperature, S_RT_, appear much smoother, lacking any uniformly distributed pattern, whereas samples deposited at higher substrate temperature, S_275 °C_, depict oval-shaped grains distributed uniformly across the surface with some regions devoid of these shapes. These images together with the XRD results confirm that elevated substrate temperature aids the improved crystallization of β-Ti_3_Au on both glass and Ti_6_Al_4_V substrates.

**Figure 3:**
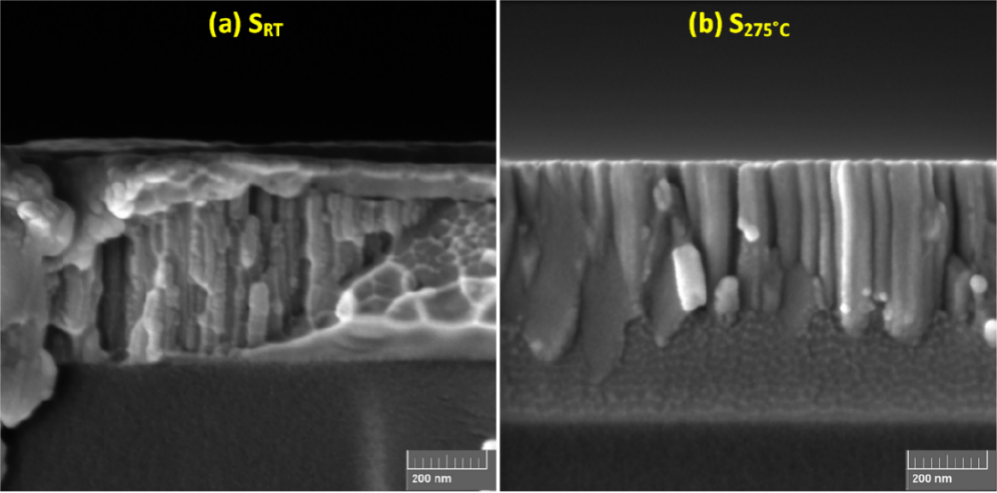
Cross-sectional imaging of Ti_3_Au thin films deposited on glass substrate at (a) room temperature and (b) substrate temperature of 275 °C.

To gain better understanding of the microstructure, the Ti_3_Au thin films deposited on glass substrates were fractured and the exposed cross-sections were characterized using SEM (see Figure 3). The sample deposited at room temperature (S_RT_) exhibits tapered columnar features extending through the partial film thickness (Figure 3a). Thornton’s structural zone model (SZM) predicts such open-voided and tapered features to be resultant of low adatom mobility on the substrate surface in the absence of substrate heating and argues that such thin films will be amorphous in nature.^23,24^ Some regions of the cross-section from this sample also exhibit vein-like patterns, characteristic of fracture propagation along amorphous thin film metallic glasses (TFMGs),^25^ thereby also supporting the amorphous nature of these thin films.^26−29^ On the other hand, the cross-section of the thin film deposited at an elevated substrate temperature of 275 °C (S_275 °C_) exhibits well-organized, dense, and broader columns with small dome-shaped surfaces (Figure 3b). Thornton’s SZM predicts that when the substrate temperature is increased, it leads to higher diffusion of adatoms along the surface as well as along the grain boundaries, which reduces the intercolumnar space, giving a dense appearance.^22^ This enhanced surface diffusion of energetic particles promotes preferred orientation growth in the columns and leads to higher crystallinity, observed as the emergence of the dominant β phase of the Ti_3_Au intermetallic in the XRD patterns seen in Figure 1.

**Figure 4:**
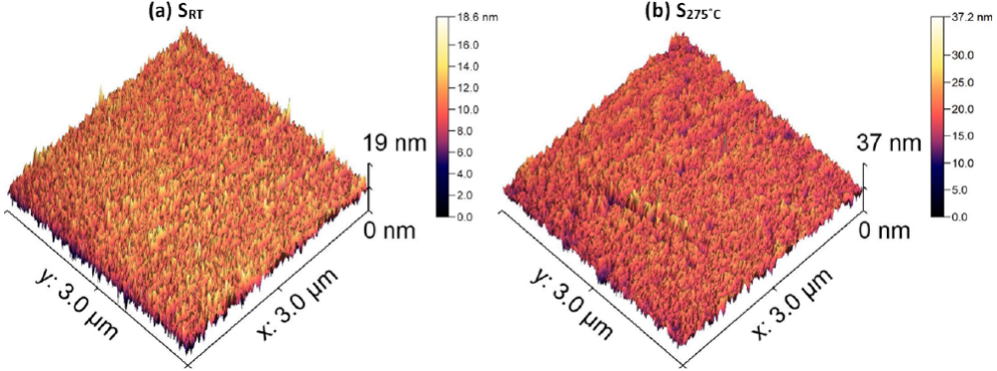
AFM scans of Ti_3_Au thin films deposited on glass substrate at (a) room temperature and (b) substrate temperature of 275 °C.

Surface AFM scans of Ti_3_Au thin films deposited on glass substrates, with and without substrate heating, are presented in Figure 4a,b, respectively. The sample deposited without substrate temperature, S_RT_, shows a very fine-grained structure, with the tallest feature sizes of around 19 nm (Figure 4a). However, the sample deposited with an elevated substrate temperature, S_275 °C_, registers a drastic increment in feature size to around 37 nm (Figure 4b). The surface roughness of the thin films, measured from the AFM scans in Figure 4, shows that sample S_RT_ has a roughness average value of 1.7 ± 0.1 nm, whereas sample S_275 °C_ registers a 2-fold increase in surface roughness to 3.4 ± 0.1 nm. This increment in surface feature height and roughness presents a measurable effect of β-Ti_3_Au phase growth taking place with an increased substrate surface temperature.

### 3.3 Mechanical Results

**Figure 5:**
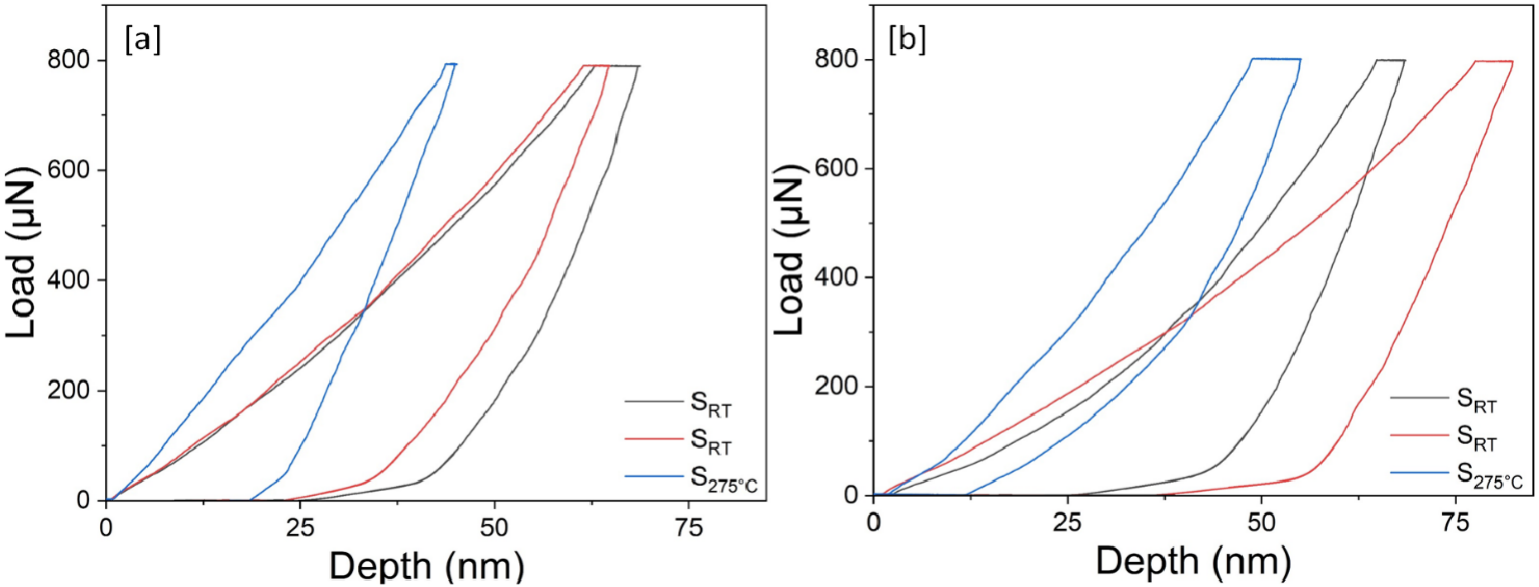
Load–displacement curves of nanoindentations made with a 800 μN load on Ti_3_Au thin films deposited on (a) glass and (b) Ti_6_Al_4_V substrates at room temperature and substrate temperature of 275 °C. All indents are at a constant 10–10–10 s load–dwell–unload segment time.

Load–displacement curves from nanoindentations made on Ti_3_Au thin films deposited on glass and Ti_6_Al_4_V substrates are presented in Figure 5a,b, respectively. To show the effect of surface roughness on mechanical testing, two examples (black and red curves) are presented from samples deposited at room temperature (S_RT_) on each substrate type, and to compare the effect of β phase growth at elevated temperature (S_275 °C_), one example (blue curve) is presented. For the sake of comparison, all of these examples are for nanoindentation performed at a peak load of 800 μN in the variable load mode. The loading and unloading segments are very smooth with no “stair step” disruption, which suggests the absence of the staircase phenomena, also known as displacement excursions.^30,31^ Such disruptions in indentation curves are normally associated with surface contamination encounter, phase transition, or oxide breakthrough events during the indentation process.^32^ If the discontinuities under the indenter do not separate, from the underlying film, no step features will appear as the film continues to support the indenter thereby preventing it from making sudden progress into the film.^33^ Depositing thin films at elevated substrate temperature rather than externally heat treating in an open furnace avoids formation of discontinuities like surface oxide layers while also providing better distribution of the β phase of Ti_3_Au, thereby achieving a smoother load–displacement curve when measuring mechanical properties using the nanoindentation technique.

**Figure 6:**
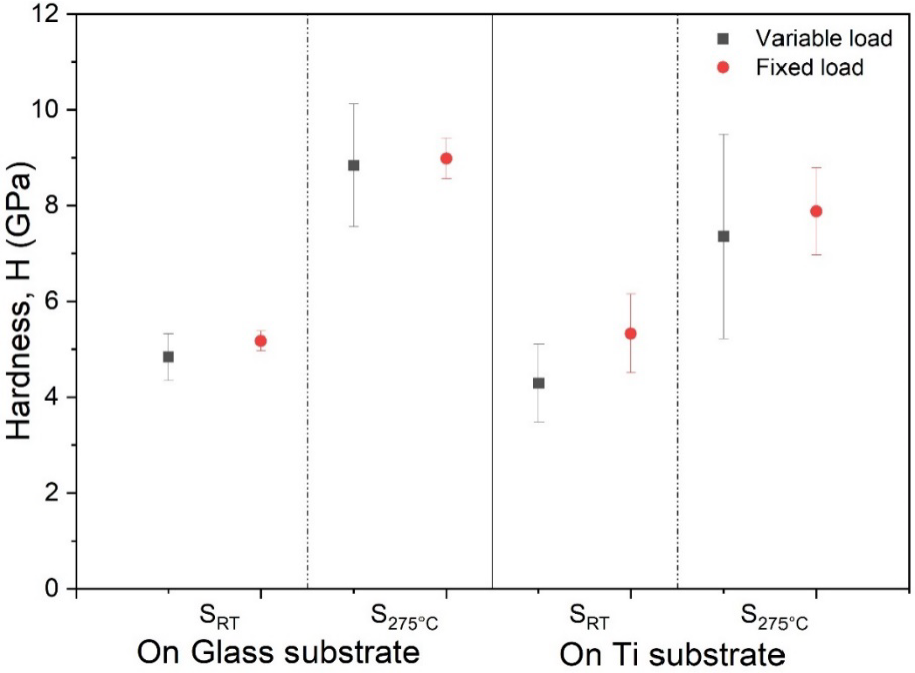
Comparison of mechanical hardness of Ti_3_Au thin films deposited on glass and Ti_6_Al_4_V substrates at room temperature and substrate temperature of 275 °C, when measured with variable load and fixed load nanoindentation methods.

**Figure 7:**
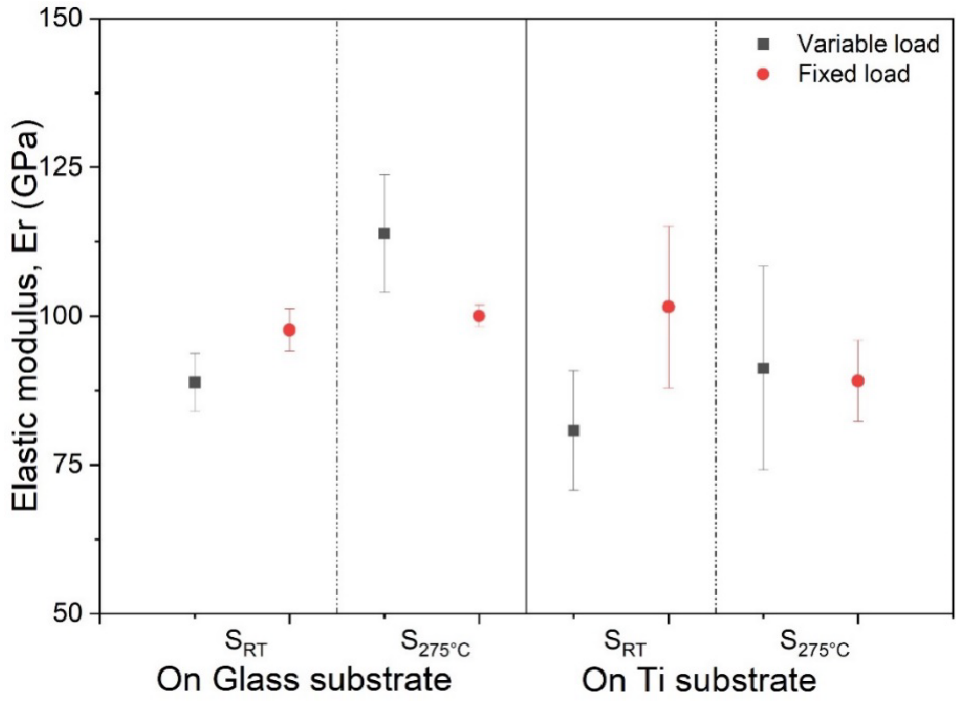
Comparison of elastic modulus of Ti_3_Au thin films deposited on glass and Ti_6_Al_4_V substrates at room temperature and substrate temperature of 275 °C, when measured in variable load and fixed load nanoindentation methods.

For indents performed on the smoother glass substrate, the loading rates of the two independent room temperature samples (S_RT_ – black and red curves in Figure 5a) look identical and the only difference arises in their unloading curve, which also looks very similar except that their trajectories give rise to a slight variation in the final indentation depth of 51 nm (red curve) and 55 nm (black curve). On the other hand, the loading rate for the two room temperature samples deposited on Ti_6_Al_4_V substrates (S_RT_ – black and red curves in Figure 5b) looks very different, even though the load specification for these indentations is identical. This variation arises because of the higher surface roughness of the underlying Ti_6_Al_4_V substrate, presenting a different topography of hills and valley-like features in the path of the approaching indenter tip and thereby affecting the loading rate in a different way each time an indent is made. This leads to a greater difference in the measured contact depth for the two samples, 64 nm (red curve) and 55 nm (black curve), and higher scatter in the mechanical results obtained. The thin film samples deposited at elevated substrate temperature, S_275 °C_, show lower indentation depth at the same peak load, suggesting that the films are becoming harder to penetrate due to the development of the β phase of Ti_3_Au. The area under the load–displacement curve represents the work done during the load–dwell–unload cycle and accounts for energy lost due to plastic deformation,^18,34^ and this mechanical hysteresis is known to decrease with heat treatment of Ti thin films due to development of crystalline phases,^18^ resulting in harder and stiffer films. These observations are also reflected in the measured mechanical properties of these thin films in Figures 6 and 7.

The hardness values of Ti_3_Au thin films deposited on glass and Ti_6_Al_4_V substrates, with and without substrate heating, are presented in Figure 6. Each of these four film samples were tested using both variable and fixed load nanoindentation techniques. In variable load mode, 16 indents were made by varying the indentation load from 2000 to 500 μN in a 4 × 4 square pattern. For the fixed load method, a second set of indents were made in a similar 4 × 4 pattern, but the load was kept constant. The fixed load required to maintain the indentation depth at a value of 10% of the film thickness was determined for each sample.^35^ It can be seen from the variable load method results in Figure 6 that sample S_RT_ deposited at room temperature on glass registers a hardness value of 4.8 ± 0.4 GPa, which increases to 8.9 ± 1.3 GPa for sample S_275 °C_, deposited at an elevated substrate temperature of 275 °C. This increase in hardness could be assigned to emergence of the superhard β phase of the Ti_3_Au intermetallic due to the elevated thermal energy of adatoms.^12,14^ When measured with the fixed load technique, the same S_RT_ and S_275 °C_ samples report similar hardness values of 5.1 ± 0.2 and 8.9 ± 0.4 GPa, respectively, but have significantly smaller deviation (error bars) when compared to the results from the variable load measurement method. On Ti_6_Al_4_V substrates, the hardness values of the S_RT_ and S_275 °C_ films reduce slightly to 4.2 ± 0.8 and 7.3 ± 2.1 GPa, respectively, and the measurement deviation increases when compared to their glass counterparts. This increased spread of results is due to the higher surface roughness (Ra < 40 nm) of the Ti_6_Al_4_V substrate when compared to much smoother (Ra < 2 nm) surface roughness of the glass substrate.^36−39^ While the thin films deposited at 275 °C are expected to report higher hardness due to better crystallization of the Ti_3_Au intermetallic, it is interesting to see that these samples also have a significantly larger spread of results compared to those deposited at room temperature, irrespective of substrate type or measurement technique. This rise in scatter could be explained by the combined effect from increasing surface roughness of the thin film at elevated substrate temperature, as seen from the AFM results (Figure 4) and the coexistence of two different phases of the Ti_3_Au intermetallic, as seen from the XRD results (Figure 1). The β phase of the Ti_3_Au intermetallic is known to exhibit higher hardness than its softer α phase, because of its denser unit cell arrangement, arising from 14-fold coordination of Ti atoms.^11,12^ This distinction between harder and softer phases of Ti_3_Au arises at higher temperatures and hence could explain the increase in the range of hardness measurements observed for thin films deposited with substrate heating.

The reduced elastic modulus values of the Ti_3_Au films deposited with and without substrate heating on glass and Ti_6_Al_4_V substrates are presented in Figure 7. The quasi-crystalline sample, S_RT_, deposited on glass reports an elastic modulus of 88 ± 5 GPa when measured with the variable load method, but with a fixed load, the same film gives a slightly higher value of 97 ± 3 GPa, with an observable reduction in the measurement scatter. For the samples deposited at elevated substrate temperature on glass, the value of elastic modulus increases to 113 ± 10 GPa in variable load mode due to development of the harder crystalline β-Ti_3_Au phase. The value remains above 100 GPa, but the error bars reduce by more than 5 times when measured with a constant load. This higher spread of results is also observed for samples deposited on Ti_6_Al_4_V substrates and, like the hardness results, can be correlated with higher substrate surface roughness, which will lead to an indenter size effect, causing larger scatter in results.^37,38^ But irrespective of substrate type, the results from both samples are more consistent around 97–101 GPa when measured with a fixed load. These values are much lower than those observed at 200 GPa in previous works for Ti–Au films deposited on Si-based substrates.^12,14^ It is known that the volume of elastic field interaction for nanoindentation tests extends much deeper than for hardness, and therefore, the values of elastic modulus are greatly affected by the underlying substrate, even when the indentation depth is maintained below 10% of the total film thickness, and this effect increases with the decrease in film thickness.^40,41^ Therefore, the lower elastic modulus of the substrates used in this work (Ti_6_Al_4_V ∼ 114 GPa, glass ∼73 GPa) explains the resulting lower elastic modulus of the Ti–Au thin films (∼113 GPa) when compared to the value of 200 GPa observed for these films deposited on Si-based substrates with higher inherent elastic modulus (Si ∼172 GPa).^40,41^ In the real world, Ti_6_Al_4_V is one of the key material systems utilized for the fabrication of artificial joint implants, and therefore, it is much more beneficial and practical to understand the behavior of superhard β-Ti_3_Au thin films deposited on this substrate system.^15^ The lower elastic modulus values of the Ti_3_Au coating material observed on Ti-based substrates are particularly important as material systems with lower elastic modulus values close to those of human bone (30 GPa) are desirous to overcome the stress shielding effect, which leads to poor bone recovery.^42^

### 3.4 Cytotoxicity–Biocompatibility Results

**Figure 8:**
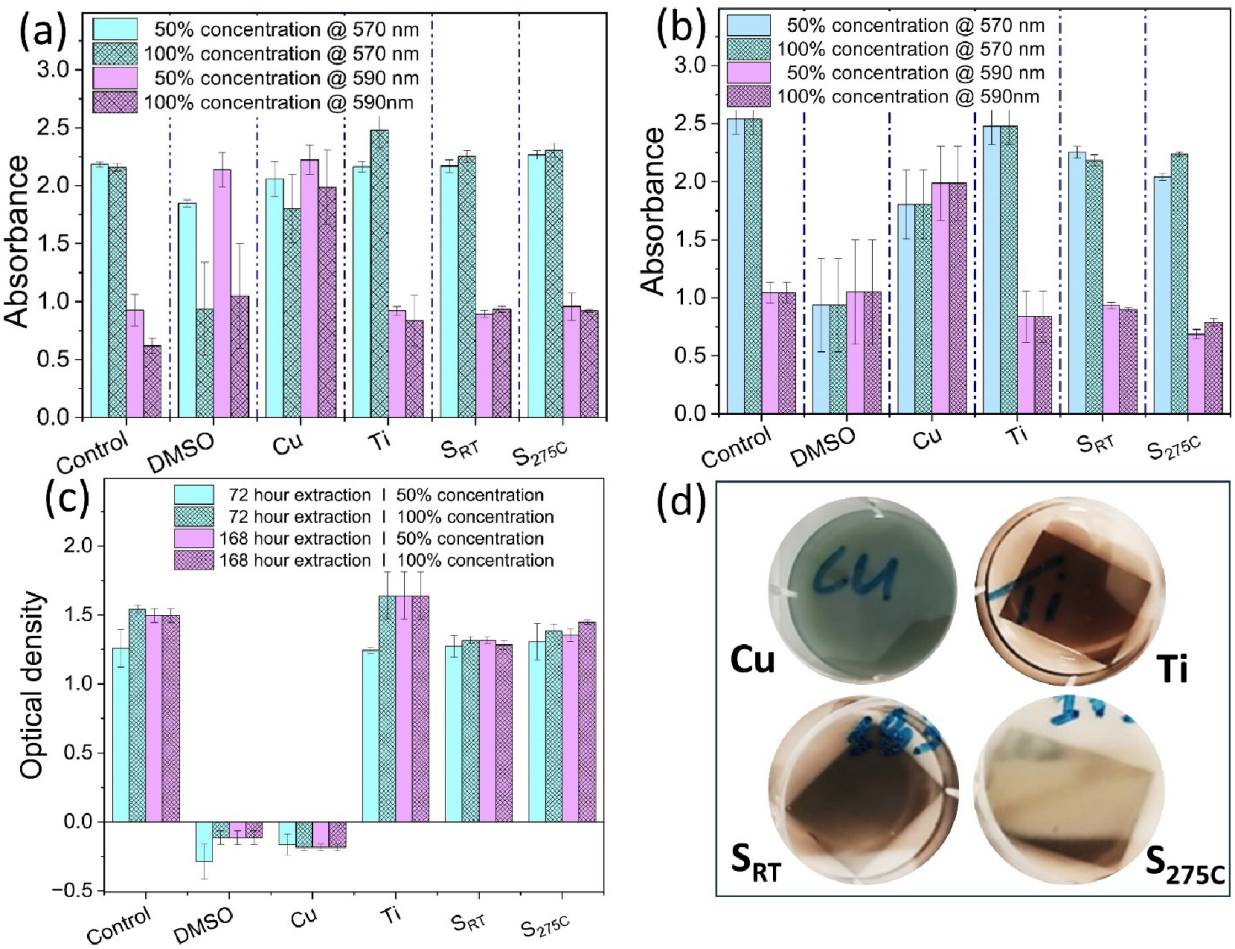
Absorbance measurements at 570 and 590 nm from L929 mouse fibroblasts exposed to film extracts for (a) 72 and (b) 168 h. (c) Optical density measured from the difference between the intensity of light absorbance at 570 and 590 nm. (d) Optical images of extracts from Cu and Ti substrates and S_RT_ and S_275 °C_ thin film samples following 168 h of extraction in DMEM culture media.

The cytotoxic effect of the different Ti_3_Au films on L929 cells was evaluated by the Alamar Blue assay. Specifically, L929 cells were incubated for 72 h with DMEM media containing leached anions obtained from 72 and 168 h of extraction from S_RT_ and S_275 °C_ thin films deposited on Ti_6_Al_4_V substrates. The extracts were tested at 100% concentration and at 50% concentration and diluted with DMEM. Similarly, L929 cells were treated with pure DMEM culture media (Control), as well as extracts obtained from 72 and 168 h of extraction from a Ti_6_Al_4_V substrate (Ti), considered as negative cytotoxic controls. On the other hand, incubations with 10% of DMSO and extracts from a Cu substrate (Cu) (obtained from 72 and 168 h of extraction) were used as positive cytotoxic controls in our experiments. Figure 8a,b shows the absorbance values of the 100% and 50% concentration extracts obtained from 72 and 168 h of extraction. It can be noticed that for known cytotoxic samples, DMSO and Cu substrate, the absorbance peak increases after the exposure as there are insufficient viable L929 cells to convert the blue-colored Resazurin reagent to purple-colored Resorufin of the assay. However, for the pure DMEM control and bare Ti substrate, which are known to be noncytotoxic, the 570 nm peak increases due to colorimetric conversion occurring within the viable L929 cells. A similar trend can be observed for the extracts prepared from the Ti_3_Au thin films (S_RT_ and S_275 °C_) deposited on Ti_6_Al_4_V substrates. The optical density values shown in Figure 8c very clearly validate this trend by plotting the difference between absorbance measurements at both wavelengths. It is seen that the known cytotoxic controls (DMSO and Cu) exhibit negative optical density values for both 100% and 50% concentration extracts obtained from 72 and 168 h of extraction, while the Ti_3_Au thin films (S_RT_ and S_275 °C_) show positive values similar to those exhibited by the known noncytotoxic DMEM and Ti controls. Figure 8d shows the change in color of the extracts following 168 h of extraction. The extract from Cu substrate appears green/bluish in color due to significant leaching of toxic Cu ions, while the extracts from the Ti substrate and Ti_3_Au thin films (S_RT_ and S_275 °C_) show no noticeable change in color, suggesting that these materials do not leach into the surrounding extract medium.

**Figure 9:**
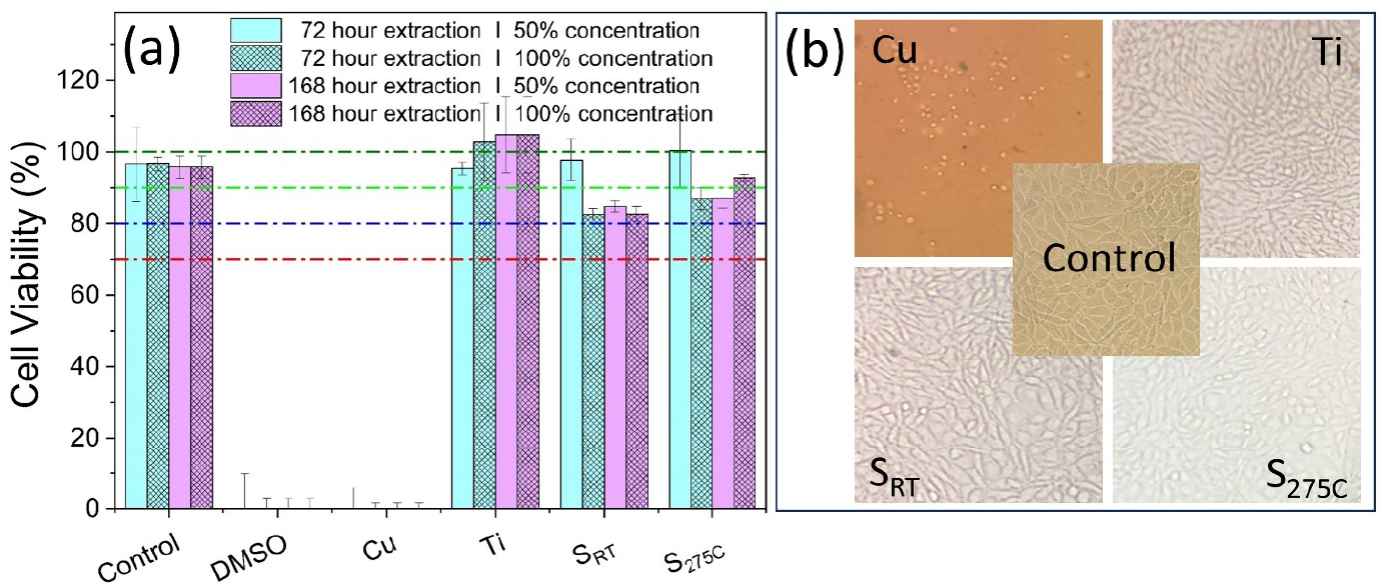
(a) Viability levels of L929 mouse fibroblast cells, following incubations with leached extracts (72 and 168 h) from S_RT_ and S_275 °C_ thin films deposited on Ti_6_Al_4_V substrates, compared against positive (Cu, 10% DMSO) and negative (Ti) controls. (b) Morphological changes of L929 cells following incubation with Cu substrate (positive control) and Ti substrate (negative control) and S_RT_ and S_275 °C_ thin film samples, compared to untreated (control) L929 cells. Images were acquired using an inverted Kern microscope with an attached digital camera and 10× lens.

Figure 9a shows viability levels of L929 mouse fibroblast cells following incubations with 72 and 168 h leached extracts from Ti_3_Au thin films deposited on Ti_6_Al_4_V substrates, compared against positive (Cu, 10% DMSO) and negative (Ti) controls. It can be seen that pure DMEM media, as well as Ti subleached extracts, have a safe cytotoxic profile, as viability levels were minimally affected, reaching values near or above 100%. On the contrary, exposures of fibroblast cells to Cu substrate extracts and 10% DMSO both caused a dramatic decrease in L929 cell viability levels, suggesting that excessive leaching of Cu ions into the extract can be as harmful as known toxic concentrations of 10% DMSO.^43^ On the other hand, all tested Ti_3_Au thin film extracts (S_RT_ and S_275 °C_), obtained from 72 and 168 h of leaching/extraction procedure, have a safe cytotoxic profile. Specifically, in the case of the S_RT_ samples, incubations with leached extracts led to a slight decrease (approximately 20%) in L929 cell viability levels, even in the case of media obtained from a prolonged leaching period of 168 h. In this context, an even better biocompatible profile was observed in the case of samples deposited at an elevated substrate temperature, S_275 °C_. Specifically, cell viability levels were observed to be 86% following incubations with 72 h leached media/extracts, while a slight improvement/increase of viability levels to 92% was seen in exposures with 168 h leached film media. According to the ISO 10993 standard, extracts registering cell viability rates above 70% after a minimum of 24 h exposures against mouse cells can be considered as noncytotoxic, indicating a potential biocompatible profile. Therefore, the above results from the Ti_3_Au thin films highlight their great potential to be safely used in biomedical applications.^44^

ICPOEMS tests were performed to measure leached ion concentrations in the Ti_3_Au thin film sample extracts but did not detect any significant elemental traces. In all the samples, the Ti concentration was found to be less than 0.1 ppm, whereas Au, Al, and V ion concentrations were below detection limits. The open void structure of the S_RT_ sample provides more surface area for ions to leach out compared to the extremely dense columnar arrangement of the S_275 °C_ sample and could therefore explain the slight reduction in cell viability for thin films deposited without substrate heating.^45,46^ In contrast, the Cu positive control had a leached Cu ion concentration greater than 112 ppm and it is well-known that Cu becomes cytotoxic above 10 ppm concentrations.^47,48^ Moreover, we have observed significant morphological modifications in L929 cells exposed to Cu substrate leached extract treatments; see Figure 9b. Specifically, Cu substrate extracts (168 h of leaching) caused shrinkage of L929 cells, dramatically reducing their confluency, thus indicating a strong cytotoxic effect. On the other hand, for incubations with Ti substrate and S_RT_ and S_275 °C_ thin film extracts (168 h of leaching), no morphological changes were observed, when compared to untreated (Control) L929 cells, confirming their potentially excellent biocompatible properties and safe cytotoxic profile.

## 4 Conclusion

This work investigated the combined mechanical and biocompatible performance potential of β-Ti_3_Au intermetallic thin films as a future coating system for the articulating surfaces of total joint implants. The Ti_3_Au thin films show quasi-crystalline nature when deposited at room temperature, but with an increase in substrate temperature to 275 °C, a mixture of α and β phases of Ti_3_Au develops. This difference is reflected in their mechanical properties, with an increase in hardness from 4 to 5 GPa for room temperature samples to 7–8 GPa for samples deposited at elevated substrate temperature. Deviation in hardness results is found to be adversely affected by increase in surface roughness of the underlying Ti_6_Al_4_V substrate and the coexistence of softer α and harder β phases and can be reduced by preferential growth of the β phase through substrate heating during deposition. Varying the indentation load also leads to substantial scatter in the results, while using a fixed load optimized to reach an indentation depth of 10% of film thickness improved repeatability of the results. The Ti_3_Au thin films are also observed to be noncytotoxic, irrespective of the deposition temperature or substrate type, with L929 cell viability levels above 80% and leached ion concentration levels lower than 0.1 ppm, following 72 h of incubation with 168 h leached extracts. Overall, this work helps to understand the effect of varying substrate type and temperature on the combined mechanical behavior and biocompatibility potential of β-Ti_3_Au thin films. Our future work will focus on further assessing the in vitro and in vivo mechanical wear resistance and biocompatibility of this unique TiAu intermetallic thin film system to help pave the way for the development of a superhard biocompatible coating material to extend the lifetime of articulating total joint implants.

## Data Availability

The data sets used and analyzed during the current study are available from the corresponding author on reasonable request.
